# Odor segmentation and identification in an abstract large-scale model of the mammalian olfactory system

**DOI:** 10.1186/1471-2202-12-S1-P188

**Published:** 2011-07-18

**Authors:** Simon Benjaminsson, Pawel Herman, Anders Lansner

**Affiliations:** 1 Department of Computational Biology, Royal Institute of Technology, Stockholm, S-10044, Sweden; 2 Department of Computational Biology, Stockholm University, Stockholm, S-11421, Sweden

## 

We integrate an olfactory epithelium and bulb (OB) model with an olfactory (piriform) cortex (OC) model, all comprising abstract graded units representing local populations of neurons, to demonstrate the overall system's applicability to odor recognition tasks. Large-scale simulations on a cluster computer allow us to evaluate an instance of the model with the size matching that of the olfactory system of a small mammal. The input data is synthesized with the intention to resemble the distribution of various features of early olfactory response patterns to naturalistic odor stimuli, e.g. [1,2].

Self-organization of the feedforward connectivity from the OB to the piriform cortex based on statistical properties of synthetic olfactory stimuli with the support of synaptic plasticity provide the capacity for generating sparse and distributed cortical representations [3]. At the cortical level, associative functions resulting from recurrent connectivity with Hebbian plasticity implement attractor memory storage and pattern processing, e.g. completion and rivalry [4]. Adaptation in cortical units, mimicking neuronal spike frequency adaptation, serves as an underlying mechanism for odor segmentation. We also investigate how backprojecting connections from the OC to the OB facilitate context dependent stimulus identification.

The model is evaluated in real-world scenarios involving odor classification and segmentation in noisy environments. Firstly, the network is trained to perform concentration invariant odor recognition. Secondly, the model identifies single-ligand components of odor mixtures it is subjected to in a testing phase. Finally, the effect of context dependence is evaluated by examining the system's capability to recognize specific low intensity stimuli when it is forced into an arousal state. Preliminary results show the dependence of performance on the network size, the number of odors and the complexity of their early olfactory activation patterns.

In a broader context, the proposed large-scale abstract models of holistic olfactory processing implements a multiscale approach to modeling olfaction. After the network has been trained, its connection matrices are utilized in simulating a more biophysically realistic model of the mammalian olfactory system (for details see poster by Kaplan et al.).
